# A cadaveric study of ulnar nerve strain at the elbow associated with cubitus valgus/varus deformity

**DOI:** 10.1186/s12891-022-05786-9

**Published:** 2022-09-01

**Authors:** Mitsuyuki Nagashima, Shohei Omokawa, Yasuaki Nakanishi, Pasuk Mahakkanukrauh, Hideo Hasegawa, Takamasa Shimizu, Kenji Kawamura, Yasuhito Tanaka

**Affiliations:** 1 Department of Orthopedic Surgery, Nara Medical University, 840 Shijo-cho, Kashihara, Nara 634-8521 Japan; 2 Department of Hand Surgery, Nara Medical University, 840 Shijo-cho, Kashihara, Nara 634-8521 Japan; 3 Department of Anatomy, Faculty of Medicine, Chiang Mai University, 50200, Chiang Mai, Thailand; 4 Excellence in Osteology Research and Training Center (ORTC), Chiang Mai University, 50200, Chiang Mai, Thailand

**Keywords:** Ulnar nerve strain, Cubitus valgus/varus deformity, Elbow flexion

## Abstract

**Background:**

Cubital tunnel syndrome can be caused by overtraction and dynamic compression in elbow deformities. The extent to which elbow deformities contribute to ulnar nerve strain is unknown. Here, we investigated ulnar nerve strain caused by cubitus valgus/varus deformity using fresh-frozen cadavers.

**Methods:**

We used six fresh-frozen cadaver upper extremities. A strain gauge was placed on the ulnar nerve 2 cm proximal to the medial epicondyle of the humerus. For the elbow deformity model, osteotomy was performed at the distal humerus, and plate fixation was performed to create cubitus valgus/varus deformities (10°, 20°, and 30°). Ulnar nerve strain caused by elbow flexion (0–125°) was measured in both the normal and deformity models. The strains at different elbow flexion angles within each model were compared, and the strains at elbow extension and at maximum elbow flexion were compared between the normal model and each elbow deformity model. However, in the cubitus varus model, the ulnar nerve deflected more than the measurable range of the strain gauge; elbow flexion of 60° or more were considered effective values. Statistical analysis of the strain values was performed with Friedman test, followed by the Williams’ test (the Shirley‒Williams’ test for non-parametric analysis).

**Results:**

In all models, ulnar nerve strain increased significantly from elbow extension to maximal flexion (control: 13.2%; cubitus valgus 10°: 13.6%; cubitus valgus 20°: 13.5%; cubitus valgus 30°: 12.2%; cubitus varus 10°: 8.3%; cubitus varus 20°: 8.2%; cubitus varus 30°: 6.3%, *P* < 0.001). The control and cubitus valgus models had similar values, but the cubitus varus models revealed that this deformity caused ulnar nerve relaxation.

**Conclusions:**

Ulnar nerve strain significantly increased during elbow flexion. No significant increase in strain 2 cm proximal to the medial epicondyle was observed in the cubitus valgus model. Major changes may have been observed in the measurement behind the medial epicondyle. In the cubitus varus model, the ulnar nerve was relaxed during elbow extension, but this effect was reduced by elbow flexion.

## Background

Cubital tunnel syndrome is the second most common peripheral entrapment neuropathy [1]. The etiology involves nerve compression by soft tissues, such as the arcuate and Osborne’s ligaments, and by bone spurs around the elbow [2, 3]. In addition, nerve overstrain and friction have been suggested to contribute to the pathophysiology of cubital tunnel syndrome [4]. Clark et al. [5] showed an 80% reduction in blood flow in the rat sciatic nerve associated with a 15% stretch. Tensile forces resulting in strains of 6‒12% in peripheral nerves have been shown to cause dysfunction and decreased perfusion in rabbits [6, 7].

Strain on the ulnar nerve has been shown to be the greatest, directly behind the medial epicondyle, at maximum elbow flexion [8]. In cubitus valgus, the ulnar nerve runs longer inside the elbow, causing ulnar neuropathy due to overtraction and increased tension [9, 10]. In cubitus varus, the triceps muscle moves anteromedially during elbow flexion, dynamically compressing and pulling the ulnar nerve, resulting in neuropathy [11, 12]. In cubitus valgus/varus, biomechanical factors may contribute to the pathogenesis of cubital tunnel syndrome.

However, how valgus/varus elbow contribute to changes of ulnar nerve strain is unknown, and the extent of deformity altering the nerve strain is unclear. Thus, this study investigated ulnar nerve strain caused by cubitus valgus/varus deformity. We created cubitus valgus/varus models using fresh-frozen cadavers, and used a strain gauge to measure the change in ulnar nerve strain at the elbow, in different positions, in each model.

## Methods

### Ethics

The study protocol involving human cadavers was approved by the Research Ethics Committee of the authors’ affiliated institutions. The cadavers used in the study were provided by the affiliated institutions. Consent to use the cadavers and submit for publication was obtained from the patients before death.

### Specimen preparation

Six fresh-frozen transthoracic specimens from five male donors and one female donor with an average age of 77 years (range, 61–95 years) at the time of death were used in the present study. The specimens were prepared by thawing overnight at room temperature one day before the experiment. The specimens included the left upper extremities, from the second cervical to the second thoracic vertebrae. The nerves running to the upper extremities maintained continuity from the spinal cord. None of the specimens had any trauma or deformity of the neck, shoulder, or upper extremity. Fluoroscopy confirmed that all specimens had no obvious elbow osteoarthritis, and the range of motion of the elbow was 0° extension and 125° flexion. The average carrying angle of the specimens was 10° (range, 0°–20°).

**Fig. 1:**
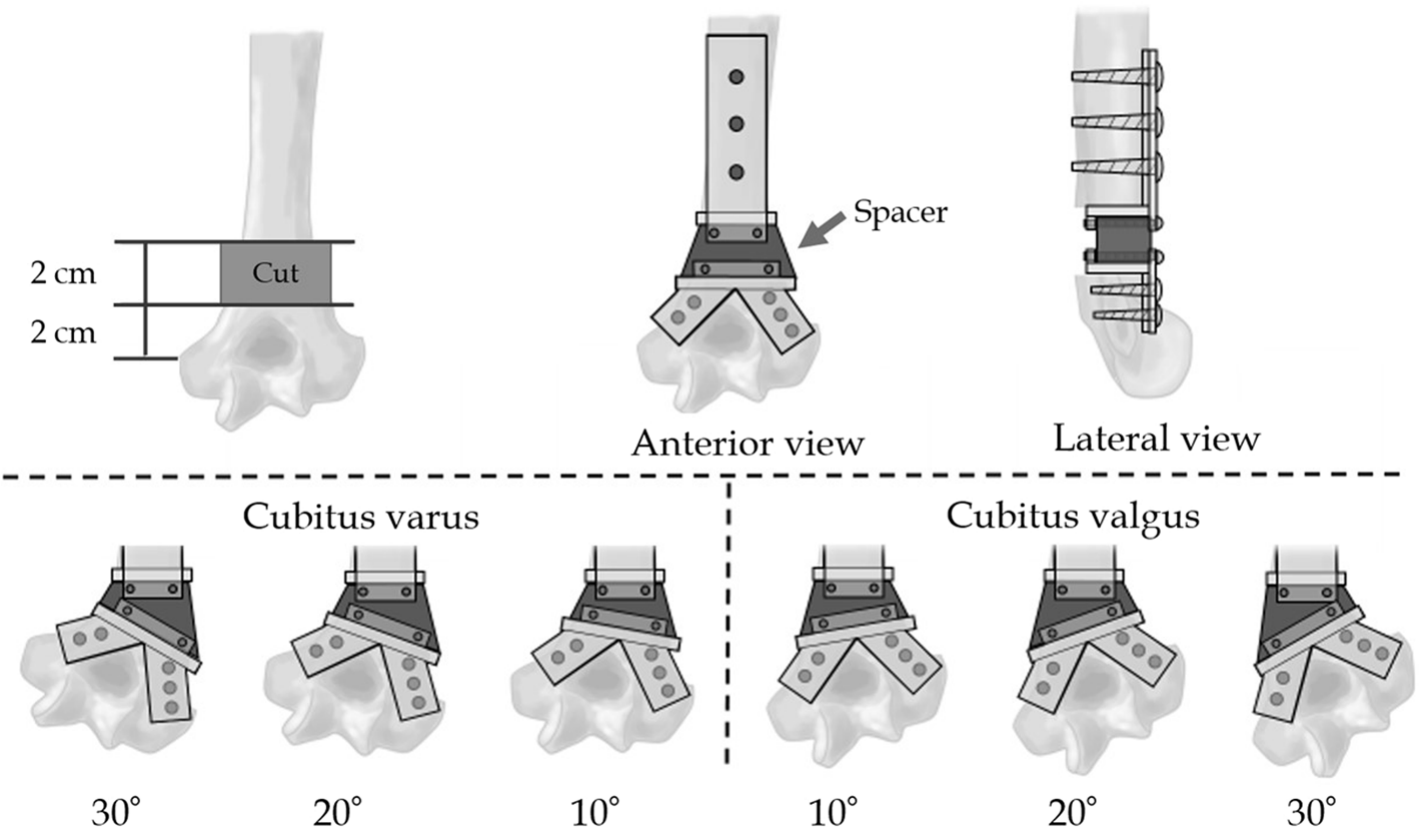
Schematic illustration of cubitus valgus/varus models created. The implant comprises proximal and distal components, which are firmly fixed to the bone, and a removable spacer. By changing the spacers, valgus and varus deformities (10°, 20°, and 30°) can be created

**Fig. 2:**
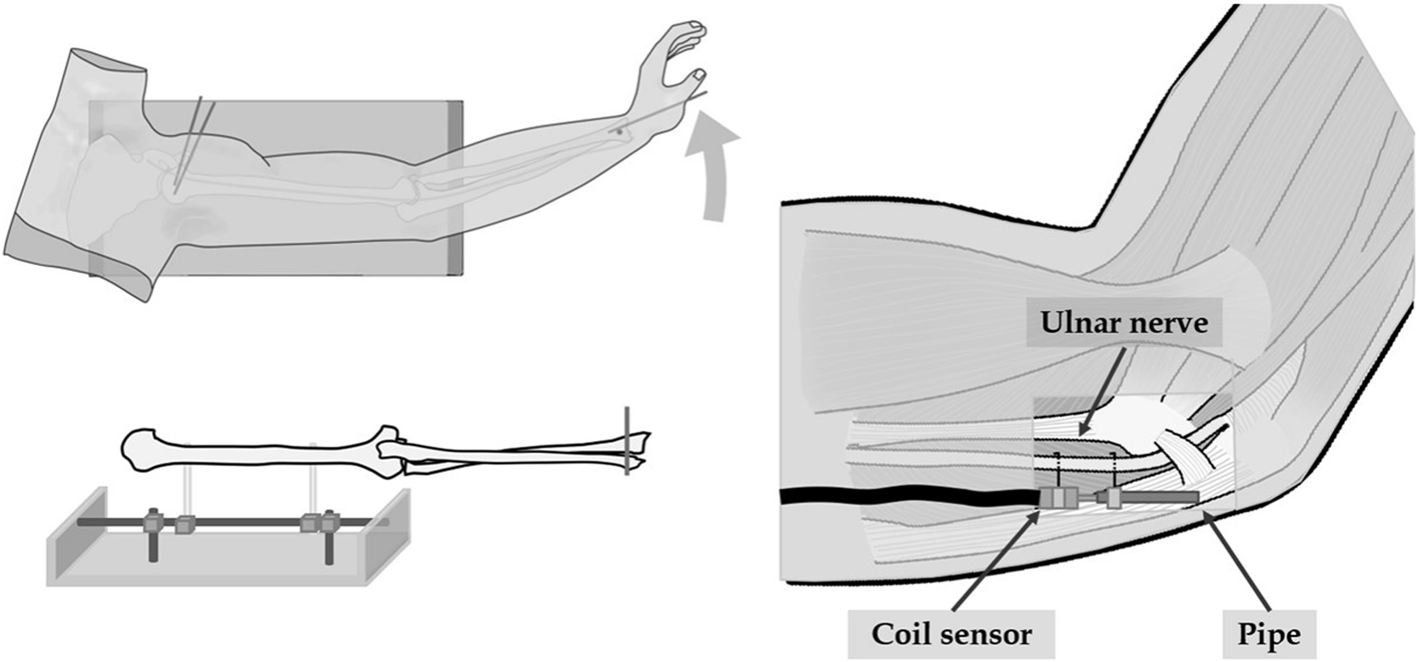
Schematic illustration of specimen fixing and strain gauge setting. An external fixator fixed the humerus to the experimental table. Two needles attached to the strain gauge were inserted into the ulnar nerve 2 cm proximal to the medial epicondyle

To create cubitus valgus/varus deformities, we used an implant with adjustable deformations. The implant consisted of proximal and distal components, which were firmly fixed to the bone, and a removable spacer. By changing the spacers, valgus and varus deformities (10°, 20°, and 30°) could be created (Fig. 1). Osteotomies were made 2 cm and 4 cm proximal to the medial epicondyle of the humerus, with an approach from the anterior side to preserve the triceps muscle, and each component was fixed anterior to the humerus. Each specimen was then placed on an experimental table with an external fixator (Fig. 2).

The ulnar nerve upper limb neurodynamic test [13] was used to determine the limb position in which the ulnar nerve was most strained. Each specimen was immobilized at 110° abduction and with 90° external rotation of the shoulder joint, maximum forearm rotation, and maximum extension of the wrist joint, using 2.0 mm diameter Kirschner wires at the shoulder, distal radioulnar, and radiocarpal joints, and only elbow motion was allowed.

### Measurement of ulnar nerve strain

Skin and fascia windows (6 cm long and 3 cm wide) were made 3 cm proximal to the cubital tunnel, and the ulnar nerve was exposed. The site of maximum nerve strain with elbow flexion is behind the medial epicondyle [8]. However, the center of rotation of the elbow deformity in our elbow deformity models is 2 cm proximal to the medial epicondyle. Moreover, in a previous study, the ulnar nerve showed the greatest strain change at 2 cm proximal to the medial epicondyle during elbow flexion except behind the medial epicondyle [14]. Accordingly, we predicted that the site most affected by elbow deformities would be 2 cm proximal to the medial epicondyle. Therefore, a strain gauge (Pulse-Coder; LEVEX, Kyoto, Japan) was placed on the ulnar nerve 2 cm proximal to the medial epicondyle, with the elbow in the extension position (Fig. 2). The strain gauge consisted of a brass pipe (32 mm long and 3 mm wide) and a rod-shaped coil sensor. The measurement system has been described previously [15]. The strain refers to the distortion (amount of elongation change) of the ulnar nerve itself that occurs as a result of the traction force applied to it. The strain gauge can measure the amount of change in ulnar nerve elongation. The measurement range was 14 mm. Twelve-millimeter-long needles, attached to both ends of the strain gauge, were inserted into the ulnar nerve at 15 mm intervals during elbow extension in the no-elbow deformity model. The needles were barbed to prevent them from slipping off the nerve. Ulnar nerve strain (%) was calculated by dividing the amount of elongation (mm, measured with the strain gauge) by the distance between the needles at elbow extension in the no-elbow deformity model (i.e., 15 mm).

### Experimental sequences

First, ulnar nerve strain was measured during elbow flexion (0°, 30°, 60°, 90°, 120°, up to a maximum flexion of 125°) in the no-elbow deformity model, which was used as the control model with an implant angulation of 0°. The elbow angles were measured using a goniometer. Six elbow deformity models were created by changing the spacers in each specimen as follows: 10°, 20°, and 30° for the cubitus valgus model, and 10°, 20°, and 30° for the cubitus varus model. The strain was measured using the same procedure. The spacer was carefully replaced while keeping the strain gauge attached. After replacement of the spacer, the skin at the entry site was sutured. The conditions were the same for all models. Each measurement was performed three times, and the average value was obtained.

### Statistical analysis

Statistical analysis of the strain values was performed with Friedman test, followed by the Williams’ test (Shirley‒Williams’ test for non-parametric analysis). Within each model, strain was compared according to the elbow flexion angle, and strains at elbow extension and maximum elbow flexion were compared between the control model and each elbow deformity model. In the cubitus varus models, the ulnar nerve deflected more than the measurable range of the strain gauge, and strains below 30° of elbow flexion were used as reference values. Consequently, the cubitus varus models were compared with the control group for elbow flexion of 60°. The level of significance was set at *P* < 0.05. Statistical analyses were performed using Statcel 4 software (OMS Publishing Inc., Tokyo, Japan).

## Results

**Fig. 3:**
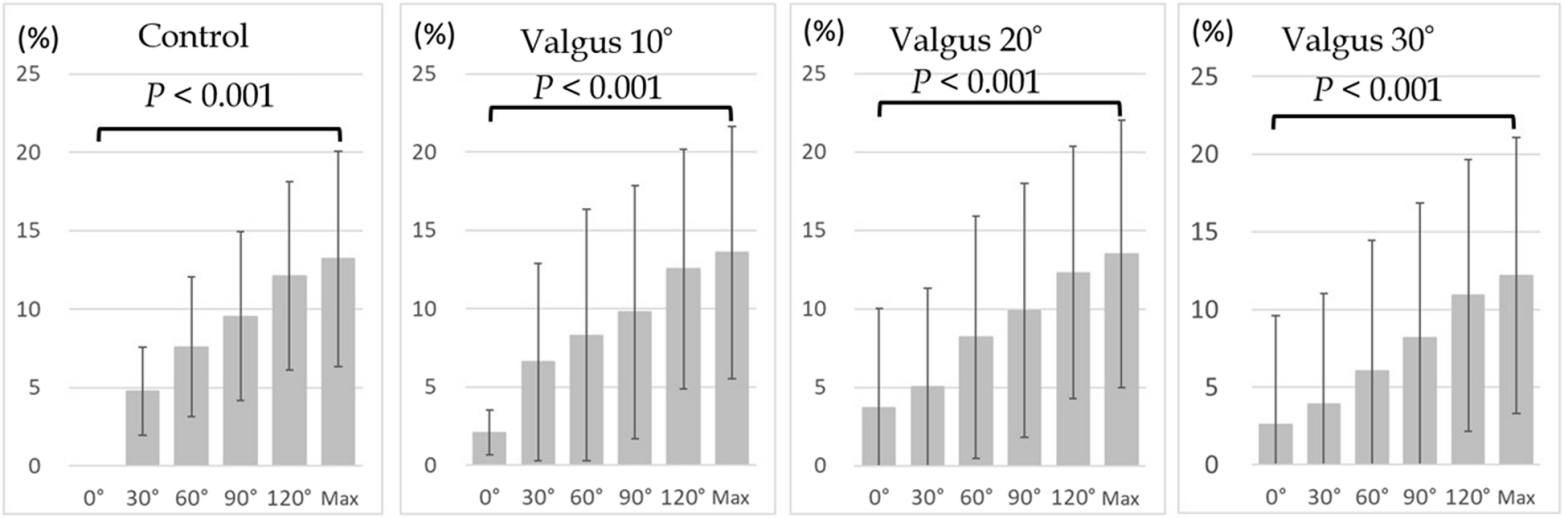
Strain on the ulnar nerve during elbow flexion in control and cubitus valgus models. *P* value indicates the significant difference in strain from elbow to maximum flexion

**Fig. 4:**
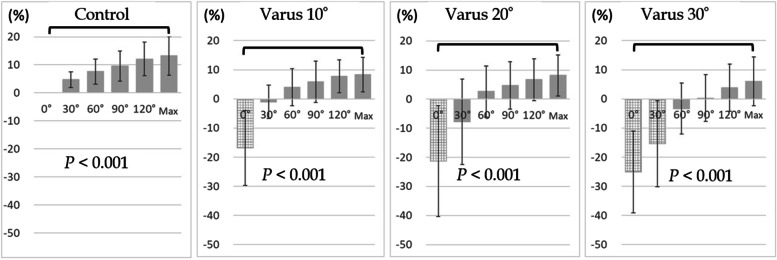
Strain on the ulnar nerve during elbow flexion in the control and the cubitus varus models. *P* value indicates the significant difference in strain from elbow to maximum flexion. The grid bar is for reference

**Fig. 5:**
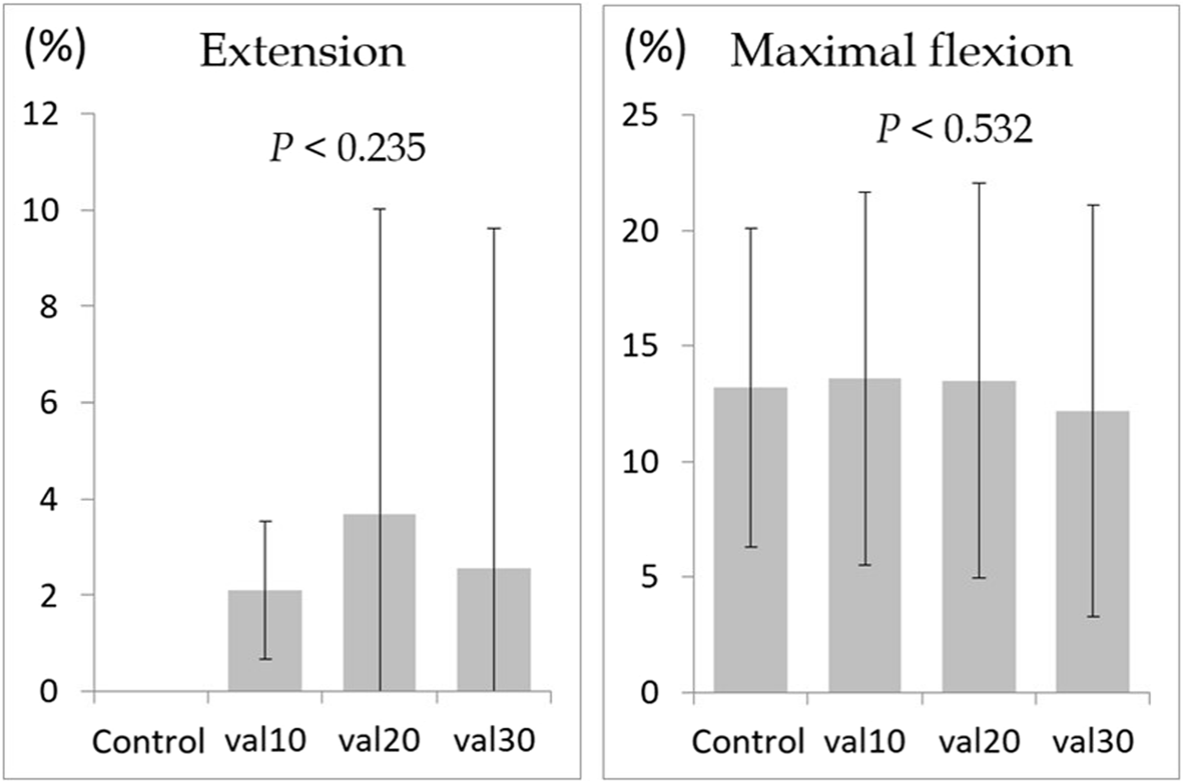
Comparison between the control and cubitus valgus models at elbow extension and maximal elbow flexion. val10: cubitus valgus 10°; val20: cubitus valgus 20°; val30: cubitus valgus 30°

In all models, ulnar nerve strain increased significantly from elbow extension to maximal flexion (control: 13.2%, cubitus valgus 10°: 13.6%, cubitus valgus 20°: 13.5%, cubitus valgus 30°: 12.2%, cubitus varus 10°: 8.3%, cubitus varus 20°: 8.2%, cubitus varus 30°: 6.3%, *P* < 0.001; Figs. 3 and 4). Comparison between the control and cubitus valgus models showed no significant difference in strain at elbow extension or maximum flexion (*P* < 0.235 and *P* < 0.532, respectively; Fig. 5).

**Fig. 6:**
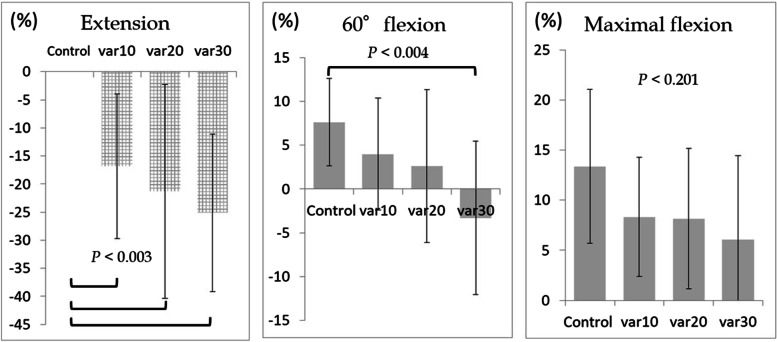
Comparison between the control and cubitus varus models at elbow extension, 60° elbow flexion, and maximal elbow flexion, respectively. The grid bar is for reference. var10: cubitus varus 10°; var20: cubitus varus 20°; var30: cubitus varus 30°

A comparison between the control and the cubitus varus models showed that the ulnar nerve was relaxed due to this deformity, and the strain was significantly reduced during elbow extension. However, these were reference values because the relaxation exceeded the measurable range of the strain gauge (*P* < 0.003; Fig. 6). The nerve strain at 60° elbow flexion was within the measurable range of the strain gauge, and the strain at 30° in the cubitus varus model was significantly less than that in the control (*P* < 0.004; Fig. 6). However, there was no significant difference in strain at maximal elbow flexion (*P* < 0.201; Fig. 6).

## Discussion

In this cadaveric study, we investigated the extent to which elbow deformities contribute to ulnar nerve strain. In all the models, ulnar nerve strain increased significantly from elbow extension to maximal flexion. The control and cubitus valgus models had similar values, but the cubitus varus models revealed that this deformity caused ulnar nerve relaxation.

The ulnar nerve glides with elbow joint movement, and the nerve itself repeatedly stretches and relaxes [4, 16]. A nerve strain of 5–10% impairs intraneural blood flow [5, 16, 17], axonal transport [18], and nerve conduction [6, 7]. Wall et al. [7] reported in their animal model that a 6% strain in a peripheral nerve for a 1 h period caused a decrease in the amplitude of the action potential, and that a 12% strain for a 1 h period caused complete conduction disturbance. Thus, overtraction of the ulnar nerve has been proposed as a contributing factor in cubital tunnel syndrome [3]. Toby and Hanesworth [8] observed that the maximum strain in this nerve was located behind the medial epicondyle during maximal elbow flexion. Wright et al. [19] found that ulnar nerve strain of 29% occurred with elbow flexion. In the current study, the ulnar nerve in the control group showed a strain of 13.2 ± 6.9% 2 cm proximal to the medial epicondyle at maximal elbow flexion, which was similar to that reported by Aoki et al. [15]: 13.1 ± 6.1% in maximal elbow flexion, 3 cm proximal to the cubital tunnel. This result showed that even in the normal elbow, elbow flexion produces significant strain. However, compliance to nerve elongation changes has been measured to be higher for nerve segments that cross the joint than for segments that do not cross the joint [20]. Therefore, we assume that nerve damage does not occur in the normal elbow.

The ulnar nerve travels a longer distance in the medial elbow in cubitus valgus, which causes nerve traction and overstrain, resulting in ulnar neuropathy [9, 10]. In the current cubitus valgus models, no significant increase in ulnar strain was observed as compared with the controls. At maximum elbow flexion, the strain was similar to that of the control. We hypothesized that ulnar nerve tension and strain would increase with cubitus valgus deformity. However, contrary to our hypothesis, we found that cubitus valgus deformity up to 30° had no effect on the ulnar nerve. We measured strain 2 cm proximal to the medial epicondyle, the center of the elbow deformity. However, maximum strain occurs behind the medial epicondyle during elbow flexion [8], therefore, measuring strain at this site may have revealed a significant change in the strain. Dilley et al. [21] found that the ulnar nerve has an undulating segment, which provides the laxity that reduces the load on the nerve caused by the flexion movement of the elbow. This section is referred to as the “high-compliance segment.” In a previous study, Nagashima et al. [14] reported that traction on the ulnar nerve caused by elbow flexion was reduced by straightening the high-compliance segment, preventing increased strain on the nerve itself. Similarly, the ulnar nerve traction caused by cubitus valgus may have been reduced by the straightening of the high-compliance segment, and thus, the strain did not increase. Therefore, increase in nerve strain may be seen in severe cubitus valgus deformity or in valgus plus rotaional deformity that exceeds the amount of stretch provided by the high-compliance segment.

In cubitus varus, the traction axis of the triceps muscle is displaced medially, causing the triceps muscle to move anteromedially during elbow flexion. The ulnar nerve is subjected to anterior subluxation due to the movement of the triceps muscle [11, 12]. This anterior displacement of the nerve results in neuropathy due to constriction by Osborne's ligament and kinking in the humeral head of flexor carpi ulnaris [22]. In the current cubitus varus models, the ulnar nerve showed significantly reduced strain with elbow extension as compared with the control, and the ulnar nerve was also visually flaccid. During maximal elbow flexion, nerve laxity was eliminated, and there was no significant difference in the nerve strain compared with the control. In our study, excessive laxity of the ulnar nerve was observed. In cubitus varus, the ulnar nerve passed a shorter distance medial to the elbow, resulting in relaxation of the ulnar nerve. This laxity may increase the short-axis movement of the ulnar nerve, facilitating hypermobile ulnar nerve with anterior subluxation [23].

Clarification of the effects of elbow deformity on the ulnar nerve may assist in the clinical management of elbow deformities. In this study, we could not detect an effect of cubitus valgus on ulnar nerve strain. Therefore, no definitive conclusion can be drawn regarding the effects of elbow deformities and we could not demonstrate the extent of acceptable clinical elbow deformity. Restriction of ulnar nerve gliding increases nerve strain by 50–154%, as compared to conditions allowing nerve gliding [24, 25]. Therefore, ulnar nerve adhesions due to trauma may be more likely to cause nerve overtraction. If nerve adhesion occurs in a way that disrupts the high-compliance segment, the nerve strain may increase with elbow deformity. An experimental model simulating ulnar nerve adhesion around the osteotomy site would increase the ulnar nerve strain in cubitus valgus deformity. In addition, pressure changes in the cubital tunnel may also be involved in ulnar neuropathy at the elbow. Additional studies are warranted to measure the pressure in the cubital tunnel and compression force at the fulcrum in cubitus valgus.

This study has several limitations. First, the sample size was small. Second, our study used frozen cadavers; therefore, it is not possible to determine the muscle contraction associated with joint motion. Caution was exercised not to damage the soft tissues, such as the paraneurium [26], which are crucial for nerve gliding during the study; however, over time, the properties of the fascia and nerves may change due to drying of the cadaver, and the environment may be different from that of in vivo experiments. Third, elbow deformity caused by trauma is accompanied by valgus/varus deformity as well as angular deformity, rotational deformity, and perineural adhesions, but these factors were not evaluated in this study. Finally, although we could measure the precise amount of strain with our sensor, we could not evaluate movement within 2 cm of the medial epicondyle because the nerve was bent with elbow flexion. Major changes may have been observed in the measurement behind the medial epicondyle.

## Conclusions

Ulnar nerve strain significantly increased during elbow flexion. Contrary to our expectations, no significant increase in strain was observed in cubitus valgus. In cubitus varus, strain decreased significantly during elbow extension due to relaxation of the ulnar nerve.

## Data Availability

The datasets generated during and analyzed during the current study are not publicly available, as additional research will be conducted in future, but are available from the corresponding author on reasonable request.
